# Deep clinical and genetic analysis of 17p13.3 region: 38 pediatric patients diagnosed using next-generation sequencing and literature review

**DOI:** 10.1186/s12920-025-02155-y

**Published:** 2025-05-19

**Authors:** Xiaoshan Ji, Qiong Xu, Yulan Lu, Bo Liu, Feifan Xiao, Qi Ni, Suzhen Xu, Renchao Liu, Gang Li, Bingbing Wu, Shuizhen Zhou, Huijun Wang

**Affiliations:** 1 Center for Molecular Medicine, Children’s Hospital of Fudan University, 399 Wanyuan Road, Shanghai, 201102 China; 2 Department of Neonatology, Children’s Hospital of Fudan University, Shanghai, 201102 China; 3 Department of Child Health Care, Children’s Hospital of Fudan University, Shanghai, 201102 China; 4 Department of Neurology, Children’s Hospital of Fudan University, 399 Wanyuan Road, Shanghai, 201102 China

**Keywords:** 17p13.3, Copy number variants, Single-nucleotide variation, Next-generation sequencing, Lissencephaly, Developmental delay

## Abstract

**Background:**

Chromosome 17p13.3 is a region of genomic instability associated with different neurodevelopmental diseases. The malformation spectrum of 17p13.3 microdeletions ranges from an isolated lissencephaly sequence to Miller-Dieker syndrome, while 17p13.3 microduplications result in autism, learning disabilities, microcephaly and other brain malformations. This study aims to provide a more comprehensive delineation of the clinical and genetic characteristics associated with 17p13.3 alterations.

**Methods:**

We retrospectively analyzed the next-generation sequencing (NGS) data of more than 40 thousand patients from January 2016 to December 2021 and identified 38 pediatric patients with copy-number variations (CNVs) or single-nucleotide variations (SNVs) in 17p13.3 region. Published patients with CNVs in the 17p13.3 region were also collected and we performed a Chi-square test to compare the phenotype spectrum of microdeletions and microduplications.

**Results:**

Among the 27 CNV patients, 20 patients with microdeletions and 7 patients with microduplications were found. *PAFAH1B1* was the most frequently deleted gene and *CRK* was the most frequently duplicated gene. Affected genes in 11 SNV patients included *PAFAH1B1* and *PRPF8.* Developmental delay was the most common abnormality detected in the 38 patients (29/38, 76.3%). Of note, Case 10 presented omphalocele and Case 23 presented scoliosis, webbed neck and bone cyst, all of which were unusual variant phenotypes in this region. The Chi-square test revealed that epilepsy, lissencephaly and short stature were statistically significant with microdeletions, while behavioral abnormalities and hand and foot abnormalities were significant with microduplications (*p* < 0.01).

**Conclusions:**

While *PAFAH1B1*, *YWHAE* and *CRK* are associated with major phenotypes of 17p13.3, *RTN4RL1* may be involved in white matter changes and *HIC1* might contribute to the occurrence of omphalocele. This study provided a comprehensive understanding of genetic information and phenotype spectrum of the 17p13.3 region.

**Supplementary Information:**

The online version contains supplementary material available at 10.1186/s12920-025-02155-y.

## Background

Chromosome 17p13.3 is a region of genomic instability characterized by high density of low copy repeats, often referred to as a “recombination hotspot” [1]. Haploinsufficiency or triplosensitivity of chromosome 17p13.3 major result in neurodevelopmental disorders [2–4].

Microdeletions of 17p13.3 are leading to neuronal migration disorders, with a spectrum of malformation ranging from isolated lissencephaly sequence (ILS) to Miller-Dieker syndrome (MDS) [4]. Both conditions are associated with lissencephaly, or smooth-brain, which leads to developmental delay (DD), intellectual disability (ID) and epilepsy. About 60% of ILS cases are associated with the haploinsufficiency of *PAFAH1B1* [5], while patients with MDS have larger deletions between *PAFAH1B1* and *YWHAE*, resulting in additional symptoms including short stature, facial deformities and variable congenital malformations depending on the size of the deletion [6].

Patients with 17p13.3 microduplications were first reported in 2009 [7] and have since drawn considerable clinical attention. Duplications of the telomeric portion containing *YWHAE* mainly manifested as overgrowth, facial malformation, DD and autism spectrum disorder (ASD). Duplications of the centromeric region containing *PAFAH1B1* caused brain malformation, microcephaly and DD [3].

Over the past decade, the advances in molecular diagnosis have made it possible to expand the genetic information associated with the 17p13.3 region. Using microarray analysis, Bruno et al. described a smaller critical genomic region to identify candidate genes for the main characteristics of microdeletions and microduplications in 17p13.3 [8]. Furthermore, Curry et al. reported the biggest cohort of 17p13.3 microduplications and largely expanded its phenotype information [3]. Here, we described 38 patients with copy-number variations (CNVs) or single-nucleotide variations (SNVs) in 17p13.3 identified through next-generation sequencing (NGS). After a literature review, we established a comparison between microdeletions and microduplications of 17p13.3 and discussed the genotype-phenotype correlation.

## Methods

### Study design and sample collection

We retrospectively collected the NGS data from patients referred to the Center for Molecular Medicine from January 1, 2016 to December 1, 2023. Inclusion criteria were as follows: (1) filtered CNVs in the 17p13.3 region identified by the CNV calling pipeline and confirmed using array-based comparative genomic hybridization (aCGH) or (2) SNVs within 17p13.3 region detected using NGS data and confirmed using sanger sequencing. Patients were excluded if they were confirmed as false positive.

Genetic tests of clinical exome sequencing (CES) or whole exome sequencing (WES) were directly requested by physicians, and genome sequencing (GS) was approved by laboratory-based physician applications. The criteria for genetic testing were approved by the ethics committees of Children’s Hospital of Fudan University (2022 − 364). Pre-test counseling was conducted by physicians, and informed consent was obtained from at least one parent of each patient. EDTA tubes were used to collect blood from the patients and available family members.

Clinical data was gathered from medical records and followed up via phone calls by clinical professionals. Within the framework of the Diagnostic and Statistical Manual for Mental Disorders Fifth Edition (DSM-5), DD is diagnosed when an individual under the age of 5 years fails to meet expected developmental milestones in several areas [9]. ID is defined as intelligence quotient (IQ) < 70 and limitations in adaptive functioning, with an onset in children over 5 years of age. Speech delay is defined as inability to demonstrate speech-language skills that is expected according to the age. Motor delay is defined as delay in sitting ( > = 8 months) or in walking ( > = 18 months). Short stature is defined as a height that is more than 2 standard deviations below or less than the 3rd percentile in the average height among children with the same ethnicity, age and sex.

### Samples were performed by NGS

At enrollment, 200µL blood was drawn from each patient for NGS analysis. Genomic DNA was isolated from blood samples, fragmented and enriched for CES using the Agilent ClearSeq Inherited Disease panel kit including 2742 genes known to cause inherited disorders [10] or WES using the Agilent SureSelect XT Human All Exon V5 kit. Sequencing was performed on an Illumina HiSeq X10 (Illumina). The detailed procedure was described in the published paper [11].

### CNV and SNV calling and verification

A CNV detection pipeline was developed based on CANOES and was combined with PhenoPro to prioritize phenotype-related genetic analysis [12, 13]. CNVs were annotated and filtered in the same way that reported in previous studies [14]. Agilent SurePrint G3 aCGH and SNP 4 × 180 K microarray (Agilent Technologies) were used to confirm the chromosomal aneuploidy or the CNVs detected by NGS following the manufacturer’s instructions. Data were processed using the DNA analytics software (Agilent Cytogenomics 4.0).

GATK best practice was employed for SNV calling [12]. Diagnosed variants were confirmed by Sanger sequencing. PCR primers were designed to amplify the target variants of the candidate gene. PCR products were sequenced, and data were analyzed using the Mutation Surveyor software (SoftGenetics). The pathogenicity of the CNVs and SNVs were evaluated according to the American College of Medical Genetics and Genomics (ACMG) guidelines [15, 16].

### Statistical analysis

Statistical analyses were performed using SPSS (26.0). Pearson’s chi-square test was used to compare the clinical features of 17p13.3 microdeletion and microduplication published in literature. The heat map was drawn using R-package ‘ComplexHeatmap’.

## Results

### Genetic findings

**Fig. 1:**
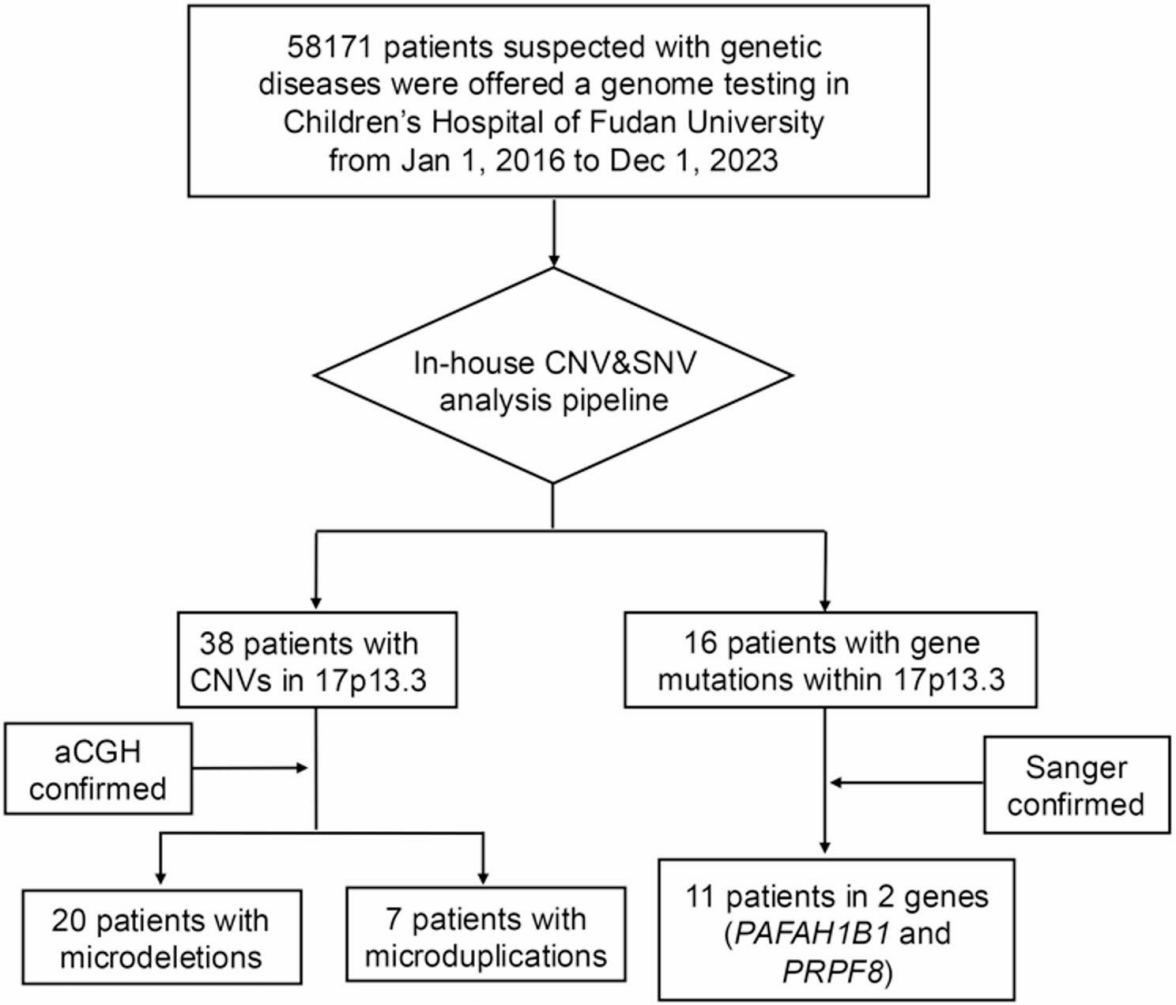
Flow diagram of this study. CNV, copy number variation; SNV, single-nucleotide variations; aCGH, array-based comparative genomic hybridization. CNV, copy number variation; SNV, single-nucleotide variations; aCGH, array-based comparative genomic hybridization

From January 1, 2016 to December 1, 2023, 58,171 patients with suspected genetic diseases were offered a genome testing (Fig. 1). A total of 54 patients with filtered CNVs based on the CNV calling pipeline and SNVs in the 17p13.3 region were enrolled in this study. Fifteen patients confirmed as false positive were excluded. A total of 27 CNVs in 17p13.3 region were included in the final analysis, including 20 microdeletions (case 1–20) and seven microduplications (case 21–27). The detailed position and size of CNVs for each patient are shown in Supplementary Table 1.

**Fig. 2:**
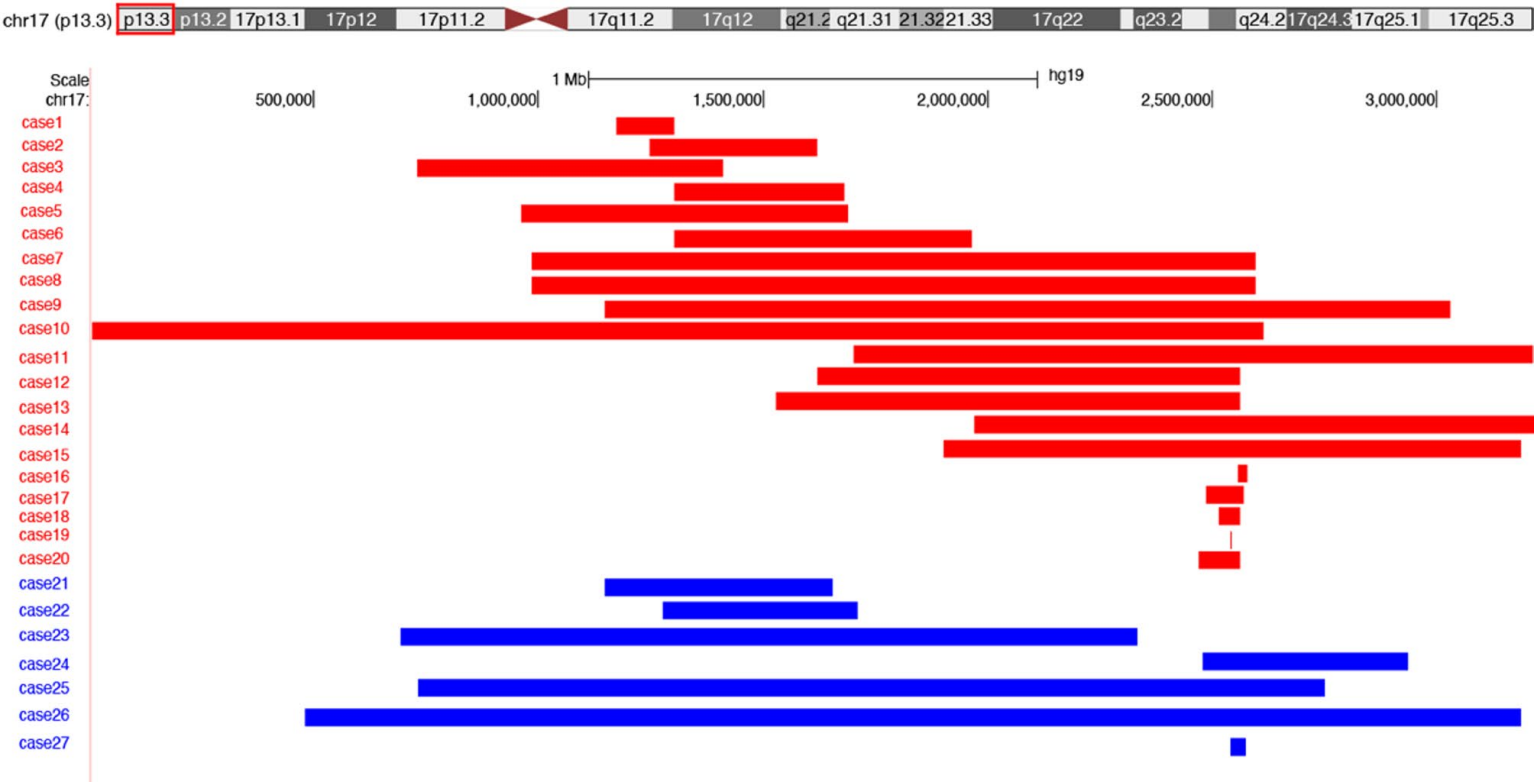
Schematic representation of 17p13.3 and summary of the molecular findings in individuals with microdeletion (red) and microduplication (blue). The figure was made using the Custom Tracks function in the UCSC Brower (http://genome.ucsc.edu)

The deletion size of the 20 microdeletion cases ranged from 20.244 kb to 5197.048 kb (Fig. 2). The biggest deletion (Case 10) spanned 2609.048 kb, encompassing 33 genes including *YWHAE* and *PAFAH1B1*. The smallest deletion (Case 16) was 20.244 kb in size and the only detected gene was *PAFAH1B1*. Briefly, the average size of losses was 886.054 kb. The most frequently deleted gene was *PAFAH1B1*, identified in 15 patients. Parental studies were available in 6 patients, and all of them were *de novo*.

The duplication size of the 7 duplication patients ranged from 34.659 kb to 2892.719 kb (Fig. 2). The largest duplication (Case 26) was 2892.719 kb. The smallest duplication (Case 27) was 34.659 kb and the only duplicated gene was *PAFAH1B1*. The average size of duplication was 1140.793 kb. Parental studies were available in 4 patients, and all of them were *de novo*.

Pathogenic SNVs were detected in 11 patients comprising 10 variants in *PAFAH1B1* and one in *PRPF8* (Case 38). Additionally, parental studies were available in 4 patients, and all of them were *de novo*. The types of variations included missense variants (5/11, 45.5%), frameshift variants (4/11, 36.4%) and stop gain variants (3/11, 27.3%).

### Clinical features

The patients’ phenotypes are detailed in Supplementary Material and summarized in Table 1. For the 20 cases of microdeletions, 14 patients (14/20, 70.0%) had DD, which was the most common neurodevelopmental abnormality. One patient (1/20, 5.0%) had ID, six patients (6/20, 30.0%) presented speech delay and 13 (13/20, 65.0%) presented motor delay. Epilepsy was observed in eight patients (8/20, 40.0%). Thirteen patients (13/20, 65.0%) showed lissencephaly and three patients (3/20, 15.0%) showed white matter abnormalities. Two patients (2/20, 10.0%) had facial malformation and three (3/20, 15.0%) were short stature. Four patients had congenital heart defects of varying severity including secundum atrial septal (case 7), ventricular septal defect (VSD, case 1 and case 10) and patent ductus arteriosus (PDA, case 5 and case 10). Omphalocele was observed in case 10, a 16-month-old girl, accompanied by congenital heart disease including VSD and PDA. Her brain magnetic resonance imaging (MRI) showed diffuse lissencephaly.

In the aspect of microduplications, six patients (6/7, 85.7%) had DD, one patient (1/7, 14.3%) had ID, five patients (5/7, 71.4%) presented speech delay, and three patients (3/7, 42.9%) had motor delay. Three patients were diagnosed with ASD (3/7, 42.9%). Hand and/or foot abnormalities were shown in two patients (2/7, 28.6%). Case 23, a 6-year-old boy, presented with the atypical phenotype of webbed neck. He also presented with mild scoliosis and a bone cyst on the palmar and radial sides of the left wrist. He exhibited small papules on the eyebrow arch and cheek, accompanied by occasional itching.

As for the 11 patients with diagnostic SNVs in *PAFAH1B1*, a total of nine patients (9/10, 90.0%) had DD, one patient (/10, 70.0%) had ID, six patients (6/10, 60.0%) had speech delay, and eight patients (8/10, 80.0%) had motor delay. Epilepsy was observed in six cases (6/10, 60.0%). Lissencephaly was observed in seven patients (7/10, 70.0%). Case 38 was a 15^+ 4^-week-old fetus whose mother carried a *de novo* mutation in *PRPF8* and suffered from retinitis pigmentosa 13. As NGS confirmed that the fetus inherited the mutation from his mother, induced labor operation was performed and the pregnancy was terminated.

### Phenotype distribution of published patients with CNVs in 17p13.3

A total of 274 cases (155 microdeletions and 119 microduplications) with CNVs in 17p13.3 have been reported [3, 6–8, 17–36]. The clinical characteristics are summarized in Table 1 and the details are summarized in Supplementary Table 2. Among the patients with microdeletions, 3.8% (3/79) were neonates and infants (< 1 year), 87.3% (69/79) were children (1 year– 18 years) and 8.9% (7/79) were adults (> 18 years). As shown in Table 1, DD (58/79, 73.4%) and epilepsy (40/78, 51.3%) were the most common neurodevelopmental disorders. Facial malformation (76/107, 71.0%) and short stature (43/70, 61.4%) were the most common structure abnormalities.

**Fig. 3:**
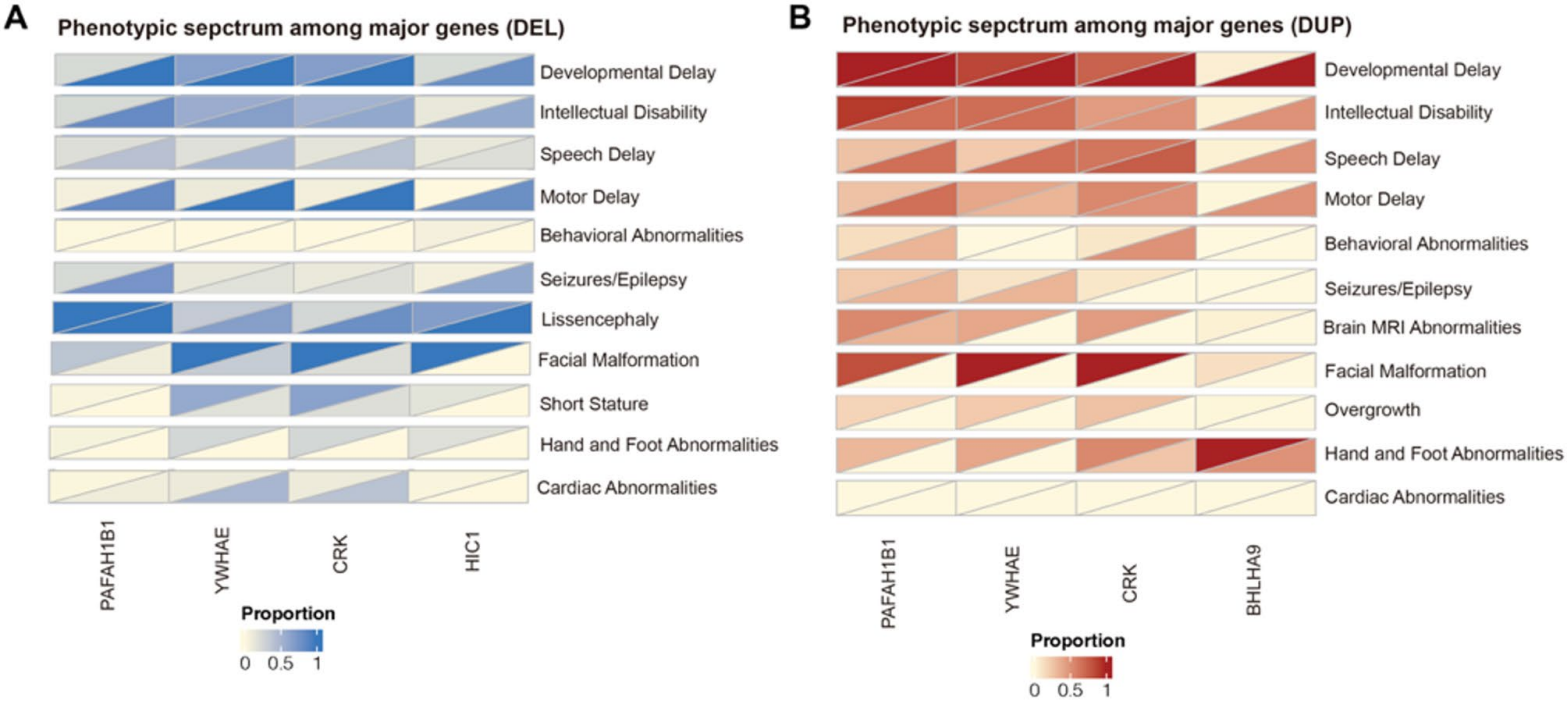
Heat map of core genes of microdeletions/microduplications in 17p13.3 among different phenotypes. The color of each cell indicated the proportion of patients with deleted/duplicated gene (row) in the relevant phenotype group (column). The upper triangle showed the genotype-phenotype correlation of our data, and the lower triangle showed the genotype-phenotype correlation collected from literature

For the reported cases with microduplication, 6.3% (4/63) were neonates and infants (< 1 year), 77.8% (49/63) were children (1 year– 18 years) and 15.9% (10/63) were adults (> 18 years). DD (66.7%, 52/78) and behavioral abnormalities (44.6%, 25/56) were on the top of neurodevelopmental abnormalities, while hand and foot abnormalities (64.4%, 76/118) were the most common structure abnormalities. The phenotype-genotype relationship of the reported cases and present cases is shown in Fig. 3.

**Table 1:** Phenotypic features of individuals with CNVs and SNVs in 17p13.3 in the present study and published studies and result of the Chi-square test

	20 microdeletions (this study) (%)	7 microduplications (this study) (%)	11 SNVs (this study) (%)	155 published microdeletions (%)	119 published microduplications (%)	χ^2^	*P*-value
**Sex**							
Male	10/20 (50.0)	4/7 (71.4)	8/11 (72.7)	59/107 (55.1)	59/108(54.6)	-	-
Female	10/20 (50.0)	3/7 (28.6)	3/11 (27.3)	52/107 (48.5)	49/108(45.4)	-	-
**Test Age**							
0–1 year 1 year– 18 years > 18 years	10/20 (50.0) 10/20 (50.0) 0/20 (0)	1/7 (14.3) 6/7 (85.7) 0/7 (0)	7/11 (63.6) 4/11 (36.4) 0/11 (0)	3/79 (3.8) 69/79 (87.3) 7/79 (8.9)	4/63(6.3) 49/63(77.8) 10/63(15.9)	- - -	- - -
**Clinical Characteristics**							
**Neurodevelopmental abnormalities**							
Developmental delay Intellectual disability Speech delay Motor delay Behavioral abnormalities Seizures/Epilepsy Lissencephaly Other brain MRI abnormalities	14/20 (70.0) 1/20 (5.0) 6/20 (30.0) 13/20 (65.0) 0/20 (0) 8/20 (40.0) 13/20 (65.0) 3/20 (15.0)	6/7 (85.7) 1/7 (14.3) 5/7 (71.4) 3/7 (42.9) 3/7 (42.9) 0/7 (0) 0/7 (0) 2/7 (28.6)	9/11 (81.8) 1/11 (9.1) 6/11 (54.5) 8/11 (72.7) 0/11 (0) 6/11 (54.5) 7/11 (63.6) 3/11 (27.3)	58/79 (73.4) 44/67 (65.6) 25/69 (36.3) 11/53 (20.8) 4/64 (6.25) 40/78 (51.3) 128/144 (88.9) 60/144 (41.7)	52/78(66.7) 43/69(62.3) 15/36(41.7) 17/37(45.9) 25/56(44.6) 7/74(9.5) 0/71 (0) 35/71 (49.3)	0.445 0.041 1.668 1.851 33.288 33.900 160.681 1.774	0.520 0.873 0.253 0.237 0.000 0.000 0.000 0.209
**Structure abnormalities**							
Facial malformation Short stature Hand and foot abnormalities Cardiac abnormalities	2/20 (10.0) 3/20 (15.0) 0/20 (0) 4/20 (20.0)	1/7 (14.3) 0/7 (0) 2/7(28.6) 0/7 (0)	2/11 (18.2) 0/11 (0) 1/11 (9.1) 0/11 (0)	76/107 (71.0) 43/70 (61.4) 33/77 (42.9) 13/73 (17.8)	46/75(60.0) 9/75(12.0) 76/118(64.4) -	0.348 31.776 17.595 -	0.555 0.000 0.000 -

CNV, copy number variation; SNV, single-nucleotide variations; MRI, magnetic resonance imaging

### Overall phenotype analysis of patients with CNVs in 17p13.3

To clarify the relationships between phenotypes of microdeletions and microduplications, we performed a Chi-square test based upon 301 patients with CNVs in 17p13.3 including 274 patients from published papers and 27 patients in this study. We found that there was no significant difference between microdeletions and microduplications in terms of DD (χ^2^ = 0.445, *P* = 0.520), ID (χ^2^ = 0.041, *P* = 0.873), speech delay (χ^2^ = 1.668, *P* = 0.253), motor delay (χ^2^ = 1.851, *P* = 0.237), and facial malformation (χ^2^ = 0.348, *P* = 0.555). However, epilepsy (χ^2^ = 33.900, *P* < 0.01), lissencephaly (χ^2^ = 160.681, *P* < 0.01) and short stature (χ^2^ = 31.776, *P* < 0.01) are statistically significant with deletions, while behavioral abnormalities (χ^2^ = 33.288, *P* < 0.01) and hand and foot abnormalities (χ^2^ = 17.595, *P* < 0.01) are significant with duplications, which was in line with gene functions.

## Discussion

In this work, we reported 38 patients with CNVs or SNVs in 17p13.3 diagnosed using NGS. Compared with other molecular detection strategies such as chromosomal microarray analysis, NGS-based approaches enable simultaneous detection of CNVs and SNVs, which can increase the diagnostic rate and reduce turn-around time and test cost.

Chromosome 17p13.3 is a region containing 90 genes, with 35 key genes that have been identified to be associated with clinical phenotypes [37]. *PAFAH1B1*, *YWHAE* and *CRK* are associated with major phenotypes of CNVs in 17p13.3, and *HIC1* and *BHLHA9* play an important role in microdeletions and microduplications, respectively. *PAFAH1B1*, *VPS53* and *PRPF8* were major affected genes in SNV patients.

*PAFAH1B1* encodes Lissencephaly-1 (LIS1) protein, which promotes neuronal migration by regulating dynein function [37]. Consistent with its function, haploinsufficiency of *PAFAH1B1* is major responsible for lissencephaly phenotypes. Our study found 18 out of 25 patients with *PAFAH1B1* deletions or intragenic variations showed lissencephaly in their MRI tests, and the lissencephaly severity were independent of the extent of deletion. Other patients with deletion or point mutation of *PAFAH1B1* showed hypoplasia of the corpus callosum, dilation of lateral ventricles and a smaller brain (case 32, 35 and 36), suggesting that LIS1 may also play a role in cell proliferation at distinct neurodevelopmental stages.

In recent years, several cases with deletions encompassing *YWHAE* and *CRK*, sparing *PAFAH1B1* have been reported. *YWHAE* encodes 14-3-3Ɛ protein that plays a regulatory role in neuronal migration and axonal growth by binding to phosphoproteins, which can explain the more severe neurological abnormalities in patients of MDS compared with ILS [38]. Mice with a deficiency of *Ywhae* have been associated with hippocampal defects, cortical thinning, defected neuronal migration and increased neuronal apoptosis [39]. *CRK*’s role in Reelin-mediated neuronal migration was also demonstrated in vivo, and it is the likely candidate for facial malformation and growth restriction [4, 40]. Five cases (Case 1–5) in this cohort carried *YWHAE* and *CRK* deletions, but not *PAFAH1B1.* The main complaint of Case 2, 3 and 4 was failure to thrive. Case 1 and 5 presented DD without MRI abnormalities, which was consistent with previous findings that the major phenotype of patients with distal 17p13.3 deletions was DD and cognitive impairment [28]. However, two patients with microdeletions encompassing *YWHAE*, sparing *PFAH1B1*, was reported recently and represented seizures as the main complaint [4, 41]. The authors proposed the possible existence of circuitry changes that were not visible at MRI imaging. Thus, *YWHAE* may play a central role in the pathogenesis of epilepsy and follow-up is necessary for our patients.

Apart from lissencephaly, white matter changes were observed in three patients (Case5, 11 and 20) in our cohort. Previous literature has considered *YWHAE* as a possible gene to explain the white matter changes observed in MDS. However, deletions in two of the patients (Case 11 and 20) did not include *YWHAE*, but included *RTN4RL1*, which encodes for NGR3, a receptor of myelin-associated inhibitors [42]. In a recent study, Emrick et al. defined an overlapping region among cases with leukoencephalopathy in 17p13.3 and speculated *RTN4RL1* may be involved in the phenotype [43]. Except these findings, the role of *RTN4RL1* to white matter changes remains largely unknown.

Behavior abnormalities and limb malformations are unique to 17p13.3 duplications. Previous studies have suggested that duplications involving *YWHAE* and *CRK* may contribute to ASD phenotypes [38]. In this cohort, 3 patients (Case 22, 26 and 27) presented ASD, with two of them carried duplications of *CRK*, and one of them carried duplications of *PAFAH1B1*. This finding suggests that the overexpression of the two genes may influence neurite formation. *BHLHA9* is known to regulate apical ectodermal ridge formation during limb development and has been implicated in split hand/foot malformations [17]. Consistent with it, Case 23 *BHLHA9* duplication presented hand malformations.

We identified two atypical phenotypes in 17p13.3 CNVs. Case 10 had omphalocele, a unique finding, accompanied by lissencephaly and congenital heart defect. This is consistent with the fact that the incidence of omphalocele is high with cardiac anomalies [44]. Given that ventral body wall defects were seen in the embryos of *Hic1-*deficient mice [45], *HIC1* may be involved in the closure of the lateral folds or the return of the midgut from the body stalk. Case 23 presented atypical phenotypes including scoliosis, webbed neck and a bone cyst on the left wrist. A Korean boy with a near-complete trisomy 17p was the only one reported to have scoliosis, but the gene contributed to the phenotype was unclear [46], and webbed neck was observed in 82% patients of trisomy 17p [47]. Given its role in cell apoptosis and webbed fingersGiven its role in cell apoptosis and webbed fingers [48], *BHLHA9* may be an attractive candidate gene for webbed neck. We need more clinical and molecular data to determine whether this is another rare phenotype of 17p13.3 duplication.

To date, 155 patients with microdeletions and 119 patients with microduplications of 17p13.3 have been reported. The distribution of neurodevelopmental disorders of each gene in our patients is less than that in the literature, while the distribution of structure abnormalities is more than that in the literature. First, the number of patients of our cohort is limited, so our result may not completely reflect the phenotype-genotype relationship of the disease. Second, the diagnostic age of our cohort was generally younger than that of the patients in previous studies. The evaluation of neurodevelopmental disorders such as ID, speech delay, and motor delay could only be performed in some patients, and behavioral abnormalities may occur later. Thus, further examinations or follow-up is needed for the younger patients in our cohort.

## Conclusions

Chromosome 17p13.3 is a region of genomic instability associated with different developmental diseases. The malformation spectrum of 17p13.3 microdeletions ranges from an isolated lissencephaly sequence to Miller-Dieker syndrome, while 17p13.3 microduplications lead to autism, learning disabilities, microcephaly and other brain malformations. We here reported 38 patients with CNVs and SNVs in 17p13.3 detected with NGS. Notably, we identified atypical phenotypes such as omphalocele and webber neck, potentially expanding the known phenotypic spectrum of 17p13.3 CNVs. The findings of this study indicate that *PAFAH1B1*, *YWHAE* and *CRK* are associated with major phenotypes of 17p13.3, and *RTN4RL1* may be involved in white matter changes and *HIC1* might contribute to the occurrence of omphalocele. Moreover, the summary of published patients with CNVs in 17p13.3 indicates that epilepsy, lissencephaly and short stature are statistically significant with microdeletions, while behavioral abnormalities and hand and foot abnormalities are significant with microduplications. Despite the increasing number of cases reported, the contribution of some genes to the phenotype is still inconclusive. Our study provides a fundamental understanding of 17p13.3-related syndromes and underscores the necessity for future mechanistic investigations.

## Electronic supplementary material

Below is the link to the electronic supplementary material.

Supplementary Material 1: Supplementary Table 1: The detailed position and size of CNVs for each patient

Supplementary Material 2: Supplementary Table 2: The detailed clinical characteristics of published patients

Supplementary Material 3: Supplementary Material: The detailed phenotypes of patients in this study

## Data Availability

All data generated or analyzed during this study are included in this article. The original data that support the findings of this study are available from the corresponding author upon reasonable request.

## Abbreviations

aCGH: Array-based comparative genomic hybridization
ASD: Autism spectrum disorder
BHLHA9: Basic helix-loop-helix family, member A9
CES: Clinical exome sequencing
CNV: Copy-number variations
CRK: CT10 regulator of kinase
GS: Genome sequencing
ILS: Isolated lissencephaly sequence
MDS: Miller-Dieker syndrome
MRI: Magnetic resonance imaging
NGS: Next-generation sequencing
p: Petit
PAFAH1B1: Platelet activating factor acetylhydrolase 1b regulatory subunit 1
PDA: Patent ductus arteriosus
PRPF8: Pre-mRNA processing factor 8
RTN4RL1: Reticulon 4 receptor like 1
SNV: Single-nucleotide variations
VPS53: Vacuolar protein sorting 53
VSD: Ventricular septal defect
WES: Whole exome sequencing
YWHAE: Tyrosine 3-monooxygenase/tryptophan 5-monooxygenase activation protein, epsilon polypeptide
