# Supplementary material for: Deep clinical and genetic analysis of 17p13.3 region: 38 pediatric patients diagnosed using next-generation sequencing and literature review
Source: BMC Med Genomics. 2025 May 19;18:90. doi: 10.1186/s12920-025-02155-y (PMC12090631; doi:10.1186/s12920-025-02155-y)
Supplement: Supplementary file 1 — Supplementary Material 1: Supplementary Table 1: The detailed position and size of CNVs for each patient [file 12920_2025_2155_MOESM1_ESM.docx]

**Supplementary Table 1**: The CNV/SNV location, CNV size and SNV variant in our 38 patients.

| Case Number | DEL/DUP/SNV variant | Location | CNV size | gene number | HI gene | Evidence | Score | Pathogenicity |
| --- | --- | --- | --- | --- | --- | --- | --- | --- |
| Case1 | DEL | chr17:1173852-1303516 | 129.664 | 3 | YWHAE | 1A(0),3A(0),4C(0.90) | 0.9 | LP |
| Case2 | DEL | chr17:1247568-1619927 | 372.359 | 15 | YWHAE | 1A(0),3A(0),4C(0.90) | 0.9 | LP |
| Case3 | DEL | chr17:722678-1401416 | 678.738 | 11 | YWHAE | 1A(0),3A(0),4C(0.90) | 0.9 | LP |
| Case4 | DEL | chr17:1303340-1680859 | 377.519 | 17 | YWHAE | 1A(0),3A(0),4C(0.90) | 0.9 | LP |
| Case5 | DEL | chr17:953289-1680859 | 727.57 | 21 | YWHAE | 1A(0),3A(0),4C(0.90) | 0.9 | LP |
| Case6 | DEL | chr17:1303340-1966535 | 663.195 | 26 | YWHAE | 1A(0),3B(0.45),4C(0.90) | 1.35 | LP |
| Case7 | DEL | chr17:975853-2588909 | 1613.056 | 44 | YWHAE, PAFAH1B1 | 1A(0),2A(1.0),3C(0.90) | 1.9 | P |
| Case8 | DEL | chr17:975853-2588909 | 1613.056 | 44 | YWHAE, PAFAH1B1 | 1A(0),2A(1.0),3C(0.90) | 1.9 | P |
| Case9 | DEL | chr17:1173852-3030875 | 1857.023 | 42 | YWHAE, PAFAH1B1 | 1A(0),2A(1.0),3C(0.90) | 1.9 | P |
| Case10 | DEL | chr17:6010-2615058 | 2609.048 | 61 | YWHAE, PAFAH1B1 | 1A(0),2A(1.0),3C(0.90) | 1.9 | P |
| Case11 | DEL | chr17:1703150-3704537 | 2001.387 | 59 | PAFAH1B1 | 1A(0),2A(1.0),3C(0.90) | 1.9 | P |
| Case12 | DEL | chr17:1648285-2588909 | 940.624 | 25 | PAFAH1B1 | 1A(0),2A(1.0),3B(0.45) | 1.45 | P |
| Case13 | DEL | chr17:1553922-2588909 | 1034.987 | 30 | PAFAH1B1 | 1A(0),2A(1.0),3B(0.45) | 1.45 | P |
| Case14 | DEL | chr17:1968761-3300000 | 1331.240 | 161 | PAFAH1B1 | 1A(0),2A(1.0),3C(0.90) | 1.9 | P |
| Case15 | DEL | chr17:1928146-3427657 | 1499.511 | 47 | PAFAH1B1 | 1A(0),2A(1.0),3C(0.90) | 1.9 | P |
| Case16 | DEL | chr17:2568665-2588909 | 20.244 | 1 | PAFAH1B1 | 1A(0),2E(0.9),3A(0) | 0.9 | LP |
| Case17 | DEL | chr17:2496947-2579900 | 82.953 | 1 | PAFAH1B1 | 1A(0),2C-1(0.9),3A(0) | 0.9 | LP |
| Case18 | DEL | chr17:2541392-2588909 | 47.517 | 1 | PAFAH1B1 | 1A(0),2E(0.9),3A(0) | 0.9 | LP |
| Case19 | DEL | chr17:2541392-2541614 | 28.999 | 1 | PAFAH1B1 | 1A(0),2C-1(0.9),3A(0) | 0.9 | LP |
| Case20 | DEL | chr17:2496947-2588909 | 92.405 | 1 | PAFAH1B1 | 1A(0),2C-1(0.9),3A(0) | 0.9 | LP |
| Case21 | DUP | chr17:1173852-1680859 | 507.007 | 19 | YWHAE | 1A(0),3A(0),4C(0.90) | 0.9 | LP |
| Case22 | DUP | chr17:1247568-1680859 | 433.291 | 17 | YWHAE | 1A(0),3A(0),4C(0.90) | 0.9 | LP |
| Case23 | DUP | chr17:662336-2304354 | 1642.018 | 43 | YWHAE | 1A(0),3C(0.90),4C(0.90) | 1.8 | P |
| Case24 | DUP | chr17:2573456-3030875 | 457.419 | 11 | PAFAH1B1 | 1A(0),2D-4(0.9),3A(0) | 1 | P |
| Case25 | DUP | chr17:685405-2703844 | 2018.439 | 54 | YWHAE, PAFAH1B1 | 1A(0),2A(1.0),3C(0.90) | 1.9 | P |
| Case26 | DUP | chr17:505034-3397753 | 2892.719 | 79 | YWHAE, PAFAH1B1 | 1A(0),2A(1.0),3C(0.90) | 1.9 | P |
| Case27 | DUP | chr17:2541392-2576051 | 34.659 | 1 | PAFAH1B1 | 1A(0),2C-1(0.9),3A(0) | 0.9 | LP |
| Case 28 | c.154delinsGA | chr17:2569346 | - | 1 | PAFAH1B1 | PVS1, PS4, PM2 | - | P |
| Case 29 | c.154delinsGA | chr17:2569346 | - | 1 | PAFAH1B1 | PVS1, PS4, PM2 | - | P |
| Case 30 | c.154delinsGA | chr17:2569346 | - | 1 | PAFAH1B1 | PVS1, PS4, PM2 | - | P |
| Case 31 | c.1219T>C | chr17:2585082 | - | 1 | PAFAH1B1 | PS2, PM1, PM2 | - | LP |
| Case 32 | c.355G>T | chr17:2570448 | - | 1 | PAFAH1B1 | PS2, PM2 | - | LP |
| Case 33 | c.405delinsGGATT | chr17:2573462 | - | 1 | PAFAH1B1 | PVS1, PM2 | - | LP |
| Case 34 | c.265C>T | chr17:2570358 | - | 1 | PAFAH1B1 | PVS1, PS4, PM2 | - | P |
| Case 35 | c.355G>C | chr17:2570448 | - | 1 | PAFAH1B1 | PS2, PM2 | - | LP |
| Case 36 | c.783G>A | chr17:2577465 | - | 1 | PAFAH1B1 | PVS1, PM2 | - | LP |
| Case 37 | c.671G>A | chr17:2576051 | - | 1 | PAFAH1B1 | PS4, PM2, PP3 | - | LP |
| Case 38 | c.6970G>T | chr17:1554134 | - | 1 | PRPF8 | PVS1, PM2 | - | LP |

Annotation: CNV, copy number variation; SNV, single-nucleotide variations; HI, haploinsufficiency; P, pathogenic; LP, likely pathogenic
