# Supplementary material for: Deep clinical and genetic analysis of 17p13.3 region: 38 pediatric patients diagnosed using next-generation sequencing and literature review
Source: BMC Med Genomics. 2025 May 19;18:90. doi: 10.1186/s12920-025-02155-y (PMC12090631; doi:10.1186/s12920-025-02155-y)
Supplement: Supplementary file 3 — Supplementary Material 3: Supplementary Material: The detailed phenotypes of patients in this study [file 12920_2025_2155_MOESM3_ESM.docx]

Case 1 was a 3-year-old girl. She presented to our hospital for developmental delay and mental retardation. She was able to walk at 2 years old and she couldn’t call her parents consciously. She was delivered after a full-term pregnancy and had a history of ventricular septal defect (VSD). She presented dysmorphic facial features including wide-set eyes and collapsed bridge of nose. Her brain MRI revealed no abnormalities.

Case 2 was a 4-year-old boy who presented to our hospital for short stature. He was delivered after a full-term pregnancy. The patient’s height was lower than that of children of the same age in the past 3 years. And the annual growth rate was 3-4 cm/year.

Case 3 was a 1-year-10-month-old boy. He presented to our hospital for growth restriction since the age of 6 months. His height was under P1 level. His speech and fine motor development were both normal for her age.

Case 4 was a 5-year-9-month-old boy. He presented to our hospital for short stature. His height and weight were 105.5 cm (-2.3SD) and 16.7kg (-1.7SD), respectively. Endocrine test demonstrated growth hormone deficiency.

Case 5 was a 1-month-old girl. She presented to our hospital for growth retardation and was diagnosed with small for gestational age. She was delivered after a full-term pregnancy and had a history of patent ductus arteriosus (PDA), pulmonary hypertension and hyperbilirubinemia. Brain MRI showed that the myelination of her brain lagged behind that compared to children of the same age.

Case 6 was a 5-month-old girl presented to our hospital for developmental delay. She was delivered after a full-term pregnancy. She was unable to raise her head up and roll over. She presented dysmorphic facial features including a prominent forehead and wide-set eyes. Her brain MRI revealed no abnormalities.

Case 7 was a 6-day-old girl. She was delivered after a full-term pregnancy and presented suffocation and groan after birth. She was diagnosed with asphyxia of newborn, respiratory failure and full-term infant with low birth weight. Echocardiography showed that she had patent ductus arteriosus (PDA). Brain MRI showed lissencephaly.

Case 8 was a 4-month-old boy presented to our hospital for developmental delay, motor delay and mental retardation. He was delivered after a full-term pregnancy. He had difficulty raising his head and presented abnormal posture in prone and supine positions. He had muscle hypotonia in upper and lower limbs. Brain MRI showed lissencephaly.

Case 9 was a 3-month-old girl. She presented to our hospital for tonic-clonic seizures three times in one day. There was a loss of awareness lasting for 2 to 3 minutes and fever was not accompanied. She also presented conjunctivitis and bronchopneumonia. Brain MRI showed that she had macrogyria.

Case 10 was a 4-day-old girl. She was born via cesarean section at 38 weeks and had a history of oligohydramnios and fetal distress. Examination of the abdomen after birth showed an omphalocele. She was transferred to our hospital for precision treatment. Echocardiography showed that she had ventricular septal defect (VSD) and patent ductus arteriosus (PDA). Brain MRI showed lissencephaly.

Case 11 was a 5-month-old boy. He presented to our hospital for generalized epilepsy for 1 month. Fever was not accompanied. Brain MRI showed lissencephaly and myelin dysplasia, possibly combined with corpus callosum dysplasia.

Case 12 was a 3-month-old girl. She presented to our hospital for developmental delay and mental retardation. She had muscle hypotonia in upper and lower limbs.

Case 13 was a 4-month-old girl. She presented to our hospital for developmental delay, motor delay and mental retardation. She was unable to raise her head. Brain MRI showed lissencephaly.

Case 14 was a 1-year-3-month-old girl. She presented recurrent seizures for a month, with eye staring, cyanosis of lip, splitting foam from the mouth, and jerks of the limbs. Each episode lasted for about 0.5 minutes. MRI showed lissencephaly.

Case 15 was a 4-month-29-day-old boy. He presented recurrent seizures for 10 days and psychomotor delay for 2 months. He had difficulty raising his head stably at the age of 3 months. He also had strabismus of his left eye. Brain MRI showed pachygyria.

Case 16 was a 4-month-old boy who was delivered after a full-term pregnancy. He presented to our hospital for new-onset generalized tonic-clonic seizures associated with loss of consciousness, lasting for two minutes. Fever was not accompanied. Brain MRI showed lissencephaly.

Case 17 was a 7-year-old girl who was delivered after a full-term pregnancy. She presented developmental delay after birth. She could roll over and grasp at 4 years old, and was unable to crawl, sit, stand and speak. She presented epilepsy at 1 year old, which occurred 2 times a day. Brain MRI showed lissencephaly.

Case 18 was a 4-year-11-month-old boy who was delivered after a full-term pregnancy. He presented developmental delay and mental retardation at 6 months old. He had difficulty raising his head stably, and was unable to roll over and grasp. There was no history of epilepsy or seizures. Physical examination showed hypotonia of the limbs.

Case 19 was a 2-year-old boy. He presented to our hospital for developmental delay and epilepsy. He was unable to roll over and grasp. He had epilepsy for 3 months, with an oral treatment of Sodium Valproate and Levetiracetam. Brain MRI showed macrogyria.

Case 20 was a 6-month-old boy who was born full-term with normal delivery and newborn course. He presented fine motor delay, with difficulty in raising his head stably, and disability to roll over and grasp. His sister had no similar symptoms. Brain MRI showed pachygyria, white matter abnormalities and enlarged ventricular system.

Case 21 was a 2-year-old girl who was delivered after a full-term pregnancy. She presented to our hospital for developmental delay and speech delay. She was not able to walk independently until 20 months old. She could only speak simple monosyllable words.

Case 22 was a 5-year-old boy. He presented to our hospital for psychomotor retardation, intellectual delay and autism spectrum disorder (ASD). He was able to walk independently at 18 months and speak simple words at 3 years old. He could follow a few simple commands. He didn’t play with his peers and was irritable, showing claw hand deformity when he lost his temper. He presented dysmorphic facial features including wide-set eyes and sparse teeth. Brain MRI showed that the left ventricle was slightly full and the corpus callosum was short.

Case 23 was a 6-year-old boy. He presented to our hospital for webbed neck, mild scoliosis and a bone cyst on the palmar and radial sides of the left wrist. He had small papules on her eyebrow arch and cheek, with occasional itchy.

Case 24 was a 2-year-old boy who was delivered after a full-term pregnancy. He presented to the hospital for global developmental delay. His developmental milestones were far behind his peers, especially in speech and fine motor. His brain MRI showed no abnormality.

Case 25 was a 2-year-old boy. He presented to our hospital for developmental delay, speech delay and motor delay, with a tendency to fall frequently.

Case 26 was a 2-year-old girl. She presented to our hospital for speech delay and motor delay. She could follow a few simple commands and speak simple words such as “mama”. She walked unsteadily and could climb stairs with support. Prenatal ultrasonography showed widened lateral ventricles.

Case 27 was a 3-year-old boy who was delivered after a full-term pregnancy. He presented to our hospital for speech delay and ASD. He had poor gaze, and was unable to speak simple words.

Case 28 was a 2-year-4-month-old boy who was delivered after a full-term pregnancy. He presented to our hospital for developmental delay. He was able to sit at 9 months old, crawl at 11 months old and walk independently at 17 months old. He could only speak simple monosyllable words. Brain MRI showed brain dysplasia and cortical thickening.

Case 29 was a 5-month-old boy. He presented to our hospital for tonic-clonic seizures, manifesting as upturned eyes, cyanotic lip and loss of conscious. He was able to raise his head at 1 month old. He couldn’t turn over or grasp. His brain MRI showed widened and deepened sulci and gyri, and an enlarged ambient cistern.

Case 30 was a 2-year-old boy. He presented to our hospital for developmental delay. He could turn over but unable to sit, stand, crawl or speak. His brain MRI showed punctate abnormal signal in the body of the corpus callosum, with slightly full anterior horns of both ventricles.

Case 31 was a 1-year-old boy. He presented to our hospital for developmental delay and epilepsy. He was unable to sit independently, and could only speak simple words such as “mama”. He had epilepsy at 6 months old. Brain MRI showed lissencephaly.

Case 32 was an 8-month-old boy who was born via cesarean section at 38 weeks and had a history of neonatal jaundice and hypoxic ischemic encephalopathy. He couldn’t grasp with his left hand at 5 months old. The movement of the right limb was not affected. Brain MRI showed brain dysplasia in the right hemisphere.

Case 33 was a 45-day-old boy. He presented to our hospital for epilepsy. He had dysmorphic facial features including wide-set eyes and long mouth. He also presented microcephaly and flared ribs. Brain MRI showed lissencephaly and slightly longer T2 signals in the medulla.

Case 34 was a 10-month-old boy. He presented to our hospital for developmental delay. he was unable to raise his head or grasp. He had no history of seizures. A 1*2 cm coffee-milk spot was found on his left arm. Brain MRI showed lissencephaly.

Case 35 was a 1-year-old girl. She was presented to our hospital for developmental delay. She presented dysmorphic facial features including a prominent forehead and wide-set eyes. Her brain CT showed hydrocephalus.

Case 36 was a 4-year-old girl. She was presented to our hospital for epilepsy for a week. The epilepsy occurred when she was awake and 3-4 times a day. The EEG showed a high degree of arrhythmia. Her brain MRI showed cortical hypoplasia.

Case 37 was a 3-year-old boy. He was presented to our hospital for recurrent seizure for 9 months. He also presented developmental delay and psychomotor retardation. Brain MRI showed lissencephaly.

Case 38 was a 15^+4^-week-old fetus whose mother carried a *de novo* mutation in *PRPF8* and suffered from retinitis pigmentosa 13. As NGS confirmed that the fetus inherited the mutation from his mother, induced labor operation was performed and the pregnancy was terminated.
